# Both whole-body rotation and visual flow induce cardiovascular autonomic response in human, but visual response is overridden by vestibular stimulation

**DOI:** 10.1038/s41598-023-31431-z

**Published:** 2023-03-14

**Authors:** O. Kuldavletova, P. Denise, H. Normand, G. Quarck, O. Etard

**Affiliations:** Université de Caen Normandie, Inserm, COMETE U1075, CYCERON, CHU de Caen, Normandie Univ, 14000 Caen, France

**Keywords:** Sensory processing, Blood flow, Respiration

## Abstract

While the influence of the vestibular and extra-vestibular gravity signals on the cardiovascular system has been demonstrated, there is little evidence that visual stimuli can trigger cardiovascular responses. Furthermore, there is no evidence of interaction between visual and vestibular signals in autonomic control, as would be expected since they are highly integrated. The present study explored the cardiovascular responses to vestibular and visual stimuli in normal subjects. We hypothesized that the visual stimuli would modify the cardiovascular response to vestibular stimulation, especially when the latter is ambiguous with respect to gravity. Off-Vertical-Axis-Rotation (OVAR) was used to stimulate vestibular and extra-vestibular receptors of gravity in 36 healthy young adults while virtual reality was used for visual stimulation. Arterial pressure (AP), respiratory rate and ECG were measured. The analysis accounted for the respiratory modulation of AP and heart rate (HR). Vestibular stimulation by OVAR was shown to modulate both mean arterial pressure (MAP) and HR, while the visual stimulation was significantly affecting HR modulation, but not MAP. Moreover, the specific visual effect was present only when the subjects were not in rotation. Therefore, visual stimulation is able to modulate the heart rate, but is overridden by vestibular stimulation due to real movement.

## Introduction

A large amount of evidence from animal and human studies suggests that vestibular input participates in autonomic response to postural change through vestibulo-autonomic reflexes^1–5^. While it has been shown that the semicircular canals, the vestibular end organs specialized in detecting head rotations, may contribute to these vestibulo-autonomic reflexes, most studies have focused on the role of the otolith organs, the vestibular end organs specialized in detecting head linear acceleration (gravity and inertial acceleration). Indeed, among the movements of the body, it is those that reorient it in relation to gravity (thus detected by the otolith organs) that mostly affect cardiovascular and respiratory systems, as they need to adjust for the changes in pressure gradient^6^. For example, it has been shown that vestibular otolith organs contribute to the control of arterial pressure (AP) during changes in posture^5^. Vestibular loss impairs cardiovascular tonic response to changes in the body orientation with respect to gravity^7,8^ or to changes in the level of gravity^9^.

Even in the absence of vestibular stimulation, visual input alone can induce a sense of self-motion, which is called vection. The role of vision in autonomic postural regulation has been much less studied than that of the vestibular system. This was first appreciated in vestibulo-lesioned cats, who were able to recover their postural AP control when visual cues for their orientation in space were present^10^. Only two studies addressed this question in human subjects, both demonstrating highly heterogeneous results. Aoki and collaborators^11^ have reported two different mean arterial pressure (MAP) adjustment reactions to illusory tilt motion induced by visual stimulus in human subjects. Six of ten subjects had their MAP significantly increased in response to vection, while four of ten decreased their MAP. Interestingly, the MAP response to real tilt was also minimal in the latter 4 subjects. Wood and colleagues^12^ also had visually induced tilt sensation in human subjects and found an increase in heart rate (HR) and a decrease in MAP in a subset of subjects. These data suggest that autonomic regulation related to gravity reorientation can be triggered by purely visual input in human subjects, albeit with high interindividual variability. In sum, the change of body orientation with respect to gravity provokes cardio-vascular adjustments, which can also be induced by purely visual tilt in some subjects.

Peripheral otolith system, as well as other extra-vestibular graviceptors, cannot differentiate between gravity and inertial linear acceleration^13^, the phenomenon known as the tilt/translation ambiguity. Convergence of visual and vestibular inputs plays a significant role in our perceptions of spatial orientation and motion^14^ and ambiguous vestibular signals^15,16^ could be disambiguated by visual input^12,17^. This raises the question of whether the vestibular and visual systems act directly on the cardiovascular system (via vestibulo- and visuo-autonomic "reflexes") or indirectly after disambiguation of the otolith input to consider only its gravitational component (*i.e.* the inclination relative to the vertical).

**Figure 1:**
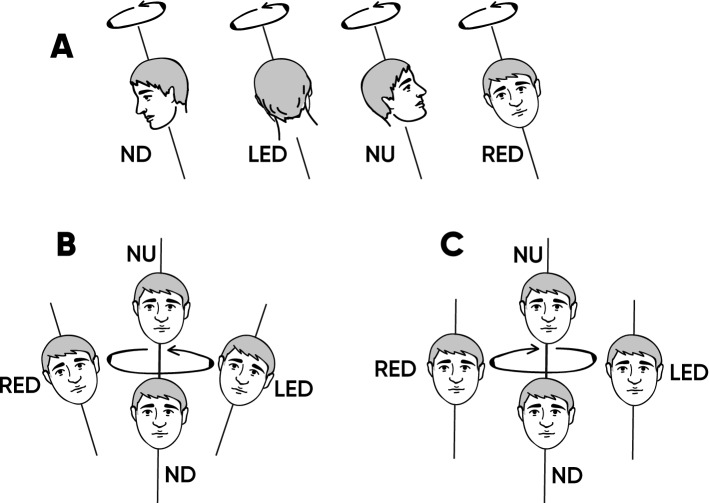
(**A**) Real OVAR movement; (**B**) Cone perception during OVAR; (**C**) Cylinder perception during OVAR. *ND* nose down, *LED* left ear down, *NU* nose up, *RED* right ear down.

To investigate whether a different visual reinterpretation of the same vestibular input could alter the autonomic response to changes in orientation with respect to gravity, we employed a protocol using combined visual and vestibular stimuli. For this purpose, we chose a perceptually ambiguous vestibular stimulus, off-axis vertical rotation (OVAR). OVAR in the dark can evoke different perceptions of self-movement (Fig. 1), most often along a conical or cylindrical pattern^16^, sometimes variations of these, like a flower-shaped pattern or inversed conical pattern^18^. The difference between the two major types of the OVAR perception (cone and cylinder) can be driven by the way the vestibular information is interpreted: as inertial acceleration (cylinder) or as gravity (cone)^19^. Our hypothesis is that during OVAR, visual input would disambiguate motion perception from the vestibular system and that the visual stimulations leading to different perceptions of self-motion, would differently affect the modulation of physiological parameters. This difference in modulation is suggested because only tilt with respect to gravity (i.e. cone perception), and not movements in the horizontal plane (i.e. cylinder perception), requires postural adjustment of the AP^5^.

According to this hypothesis our predictions were that (1) the vestibular stimulation would modulate AP and HR; (2) the visual stimulation would lead to a partial reinterpretation of the same vestibular information (OVAR): combined to OVAR, the visual stimulation that would partly be interpreted as tilt (“Cone”) would provoke a greater modulation than the one that is entirely interpreted as translation (“Cylinder”).

## Materials and methods

### Participants

48 healthy adult volunteers were recruited for this study. Prior history or clinical evidence of vestibular and neurologic disorders were the criteria of exclusion. Subjects who have not finished the protocol on the rotatory chair due to motion sickness (9 subjects: 5 females, 4 males) or technical issues (3 subjects) were excluded from the final analysis. Therefore, data from 36 subjects (15 females, 21 males; 24 ± 5 years old) were reported. Informed consent was obtained from all participants. The study was approved by an Ethics Committee (CPP Ouest I n° IDRCB 2022-A01513-40). All methods were carried out in accordance with relevant guidelines and regulations.

### Stimulation

OVAR was performed using an automated rotatory chair driven by a MicroFlex E150 servo drive (ABB, Zurich, Switzerland) operated with Mint WorkBench software (ABB, Zurich, Switzerland), with an axis inclined at 10° with respect to gravity. The direction of rotation was counterclockwise. The velocity of rotation was 60°/s (0,167 Hz), which is perceptually the most ambiguous frequency for vestibular cues interpretation, according to previous studies^20,21^. This frequency was chosen to facilitate switching, by visual stimulation, between cone and cylinder movement perception.

Visual stimulation was presented with virtual reality (VR) googles (HTC Vive, angle 110°, 90 Hz, 1080 × 1200 pixels per eye). The virtual environment represented a room with furniture to give reliable visual cues for verticals. The rotation of the virtual visual environment was synchronized with the rotation of the chair with the help of an integrated accelerometer, so the visual motion velocity was also 60°/s. Two visual motion patterns were created: moving along a Cylinder with a radius about 1 m or a Cone with a radius about 0.6 m, an apex below the body and a 10° inclination with respect to the gravitational vertical. The cylindrical motion pattern was created as a sum of harmonic oscillations along the interaural and naso-occipital axes. The conical path was composed of the same oscillations, but of lower amplitude, with the addition of periodic tilt around the same axes. Thus, the Cylinder is a translation-only movement in a plane parallel to the floor, while the Cone is the combination of the translation and tilt with respect to vertical (Fig. 1).

### Measurements

Continuous arterial pressure, as well as systolic—SAP, diastolic—DAP and MAP, were measured with an automatic continuous noninvasive arterial pressure monitor (Finometer Pro, FMS, Amsterdam, The Netherlands) placed on the middle finger of the left hand held at the heart level. The correction of hydrostatic levels was applied with the help of a Finometer integrated system. Pressure values provided by this system do not significantly differ from those taken directly from the radial artery in subjects in various physiological conditions^22^, and there is a good agreement in the evaluation of beat-to-beat variations^23^. Electrocardiogram (ECG) was measured with a 3-lead ECG (Contact Precision Instruments, Cambridge, USA). Breathing frequency (BF) was measured with the help of a commercial monitor and nasal prongs (Oridion, Microcap monitor CO2, Jerusalem, Israel). The parameters analyzed were the RR-interval (RRI, ms), MAP, SAP, DAP (Torr), and BF (min^−1^). A one-axis accelerometer (FA101, FGP Sensors & Instrumentation, France) was used to record the phases of the chair rotation.

All signals were acquired with the help of the acquisition system Notocord-hem (Notocord Hem 4.2, Notocord Systems, Croissy-sur-Seine, France).

The level of MS evoked by the rotatory chair stimulation was assessed before and after the experimental session with diagnostic criteria proposed by Graybiel and colleagues^24^. The symptoms were divided into categories with varying symptom intensities. The subject could underline only one level of intensity per category. 

### Procedure/experimental design

Subjects sat on a rotatory chair in darkness. The subjects were secured with a harness and a head support restrained the movements of their heads while rotating on the chair to prevent the cross-coupling stimulation. During all the experimental trials, they wore VR goggles, except for the pauses. Every subject performed 6 trials combining 3 visual conditions: (1) self-rotation along a cylindrical path, i.e. Cylinder, (2) self-rotation along a conical path, i.e. Cone and no visual stimulus, i.e. Darkness with 2 vestibular conditions: (1) OVAR and (2) motionless. During the Motionless condition, subjects sat on the rotatory chair without any physical motion applied. During the OVAR condition, the rotation started with the axis of rotation inclined, in the dark. The duration of every trial was 114 s, followed by a pause. The visual stimulation was turned on after 1 min of OVAR, in order to minimize the time the subjects experienced the combination of visual stimulations with actual rotation which was relatively nauseogenic. The OVAR and Motionless trials were interchanging, in order to diminish the possibility of motion sickness provoked by OVAR. The order of the visual and visual-vestibular trial pairs was counterbalanced between subjects. The MS diagnostic criteria were applied twice: before and after the experimental session and the difference between the scores after and before was taken as the final MS score. The score of 1–2 points was interpreted as Slight Malaise, 3–4 points as Moderate Malaise A, 4–7 points as Moderate Malaise B, 8–15 as Severe Malaise and ≥ 16 points were regarded as Frank Sickness.

### Analysis

**Figure 2:**
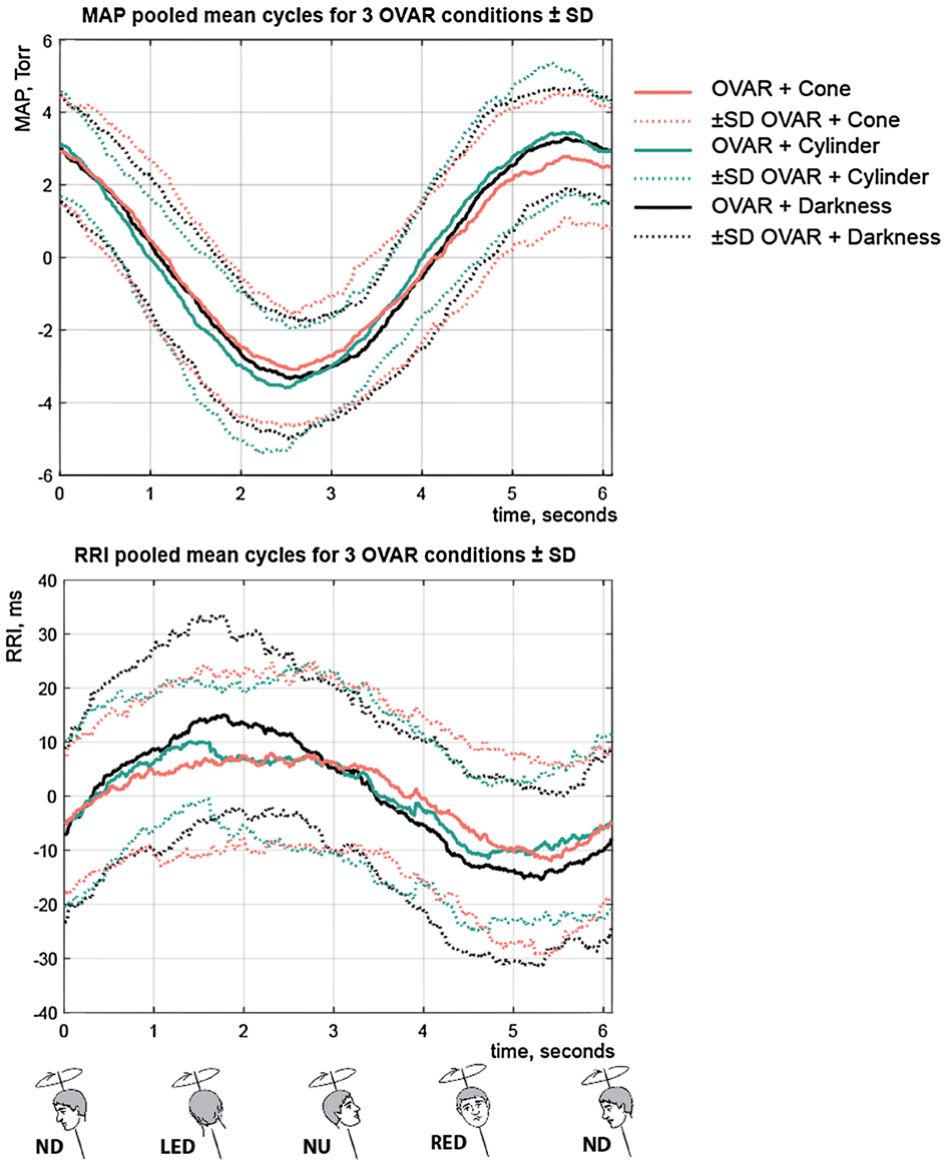
Grand mean of MAP (left) and RRI (right). Nine cycles of a parameter’s modulation by OVAR were averaged across all cycles and for all subjects for three OVAR conditions: rotation in darkness (black), rotation with the visual Cone movement delivered with the virtual reality system (red) and rotation with the visual Cylinder movement delivered with the virtual reality system (turquoise). Dotted lines delimit the standard deviation for each point of the cycle. MAP and RRI are normalized by subtracting the mean. *ND* nose down, *LED* left ear down, *NU* nose up, *RED* right ear down.

**Figure 3:**
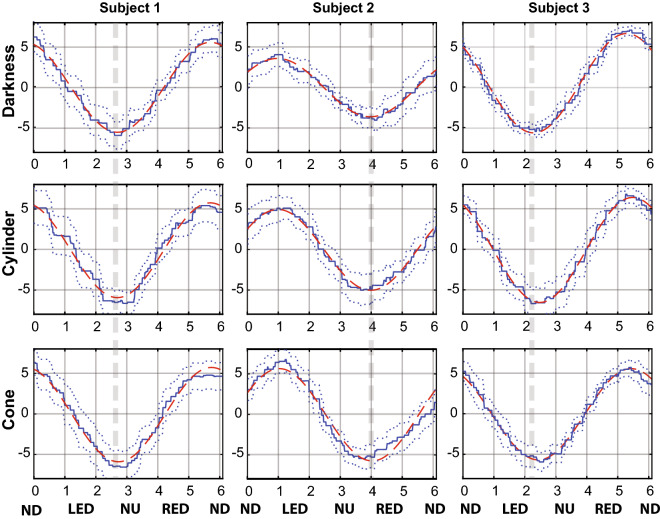
An example of an averaged cycle across 9 cycles of modulation of MAP at the stimulus frequency for 3 subjects and 3 conditions with rotation. The averaged cycle is depicted in blue with SD in dotted blue. The fitted cosinor model is depicted in red. Grey dotted lines highlight individual phase of modulation of MAP that is stable across conditions. MAP is normalized by subtracting the mean. *ND* nose down, *LED* left ear down, *NU* nose up, *RED* right ear down.

The first 60 s of the recording were discarded to assure that the vestibular stimulation is purely otolithic, without the semicircular canals’ component. The recordings were cut cycle by cycle, based on the accelerometer data, and 9 cycles were averaged for every condition and for every subject. The grand mean—the cycle averaged across all cycles and all subjects for the three visual conditions during OVAR is shown in Fig. 2. An example of individual mean cycles for the three conditions is shown in Fig. 3. A cosinor model was fitted to these averaged cycles, and offset and modulation (amplitudes and phase) were estimated. As breathing is known to modulate the HR through a mechanism called respiratory sinus arrhythmia, we have calculated the frequency at which the spectral peak of the BF occurred, in order to control for this modulation predictor. The operations were effectuated in Matlab R2017a.

### Statistics

Average values of the MAP, SAP, DAP, RRI and breathing rate were compared with RM ANOVA with a condition factor (6 conditions). In order to test the influence of rotatory and visual conditions on the modulation of MAP and RRI and their synchronization by breathing, we ran a linear mixed model analysis in R (R Core Team, 2021, Boston, MA, USA) using the linear mixed-effects models fit by maximum likelihood from the *lme4* package^25^. The p-values were provided with *lmerTest* package^26^. Pairwise contrasts were made with *multcomp* package^27^ between the sessions with the Dunnett method and FDR method p-value adjustment. To assure the normality of residuals, the amplitude of modulation of RRI (RRI_A_) was transformed to the natural logarithm of RRI_A_. To model the effect of stimuli and respiration on the modulation of RRI and MAP we included natural logarithm of the RRI_A_ and the amplitude of modulation of MAP (MAP_A_) as dependent variables and added fixed effects of mean breathing frequency, rotatory condition (OVAR/Motionless), visual condition (Darkness/Cylinder/Cone), as well as the interactions between rotation and visual stimulation. We included Subject as a random effect. The model specification for MAP modulation was as follows: *MAP*_*A*_ ~ *Breathing Frequency* + *Rotation* + *Visual* + Rotation * Visual + *(1|Subject),* the same for the natural logarithm of RRI_A_*.* The effect of the duration of the experiment on RRI, MAP, RRI_A_ and MAP_A_ was tested with the RM ANOVA with an order factor (6 trials). Comparisons of the average RRI, average MAP, RRI_A_ and MAP_A_ between “motion sick” and “not motion sick” subgroups were made with the Mann–Whitney test.

## Results

The MAP, SAP and DAP, the RR interval, and the breathing rate averaged through 54 s of measurements across all the subjects for each condition are presented in Table 1. RM ANOVA with a condition factor (6 conditions) has shown no statistically significant differences for mean, systolic and diastolic arterial pressure and the RR-interval between conditions. Breathing rate was found significantly different with RM ANOVA (p = 0.045 with a Huynh–Feldt sphericity correction), however, the Bonferroni-adjusted post-hoc test has shown no significant difference between any of the conditions.

**Table 1:** The mean values for the arterial pressure, RR interval and breathing rate.

	MAP, Torr	SAP, Torr	DAP, Torr	RRI, ms	BR, rpm
All conditions pooled	91.0 ± 11.4	117.8 ± 13.5	73.1 ± 10.8	808.5 ± 146.7	15.6 ± 3.4
Darkness motionless	90.0 ± 10.4	118.2 ± 13.2	74.4 ± 9.9	825.9 ± 149.9	14.6 ± 2.7
Cylinder motionless	90.2 ± 11.1	116.8 ± 13.0	72.8 ± 10.9	812.6 ± 137.9	15.4 ± 4.0
Cone motionless	90.5 ± 12.0	116.7 ± 13.5	73.0 ± 11.2	798.9 ± 136.3	14.9 ± 3.1
Darkness OVAR	92.5 ± 11.4	120.2 ± 13.5	74.0 ± 11.1	815.7 ± 164.8	15.7 ± 3.5
Cylinder OVAR	91.2 ± 11.4	118.9 ± 13.9	72.9 ± 10.6	789.1 ± 149.8	16.2 ± 3.7
Cone OVAR	89.8 ± 12.3	116.1 ± 14.2	71.8 ± 11.7	808.9 ± 147.5	16.4 ± 3.4

Figure 2 shows the grand mean of the modulation cycle of the MAP and RRI at the stimulus frequency, averaged across all the participants for the three OVAR conditions. On average, there seems to be no difference in the amplitude or phase of the modulation neither for MAP, nor for RRI between conditions. A subject-by-subject visual inspection suggests that the phase of the modulation is quite stable for every subject in the three rotational conditions. However, this phase differs from subject to subject, which is quite visible for the MAP (Fig. 3).

Modulations of the MAP and RRI at the frequency of the body rotation induced by visual and/or vestibular stimulation are evaluated with the mixed models.

For MAP_A_ (Table 2) there were significant main effects of respiratory rate and rotation, indicating that the MAP_A_ modulation is affected by breathing frequency and by rotation. For RRI_A_ (Table 2) there were significant main effects of breathing frequency and rotation, and also of the visual Cone effect, which seems to be different depending on the rotation.

**Table 2:** Summary of the LMM model evaluating the effect of the breathing frequency, rotation and visual stimulation, and the interaction between rotation and visual stimulation on the amplitudes of modulation of MAP and RRI (in the logarithmic scale).

	MAP_A_					RRI_A_				
	Estimate	SE	DF	t-value	p-value	Estimate	SE	DF	t-value	p-value
(Intercept)	0.27	0.25	174	1.08	0.2811	3.33	0.31	174	10.63	< 0.0001***
Breathing frequency	− 3.83	0.92	174	− 4.14	0.0001***	− 5.43	1.15	174	− 4.72	< 0.0001***
OVAR	1.99	0.13	174	15.36	< 0.0001***	0.76	0.15	174	4.98	< 0.0001***
Cylinder	0.03	0.13	174	0.24	0.8124	0.20	0.15	174	1.28	0.2013
Cone	0.08	0.13	174	0.61	0.5453	0.44	0.15	174	2.87	0.0047**
OVAR: Cylinder	0.02	0.18	174	0.13	0.8991	− 0.27	0.22	174	− 1.24	0.2159
OVAR: Cone	− 0.17	0.18	174	− 0.93	0.3517	− 0.47	0.22	174	− 2.19	0.0300*

Signif. codes: ‘***’—p < 0.001, ‘**’—p < 0.01, ‘*’—p < 0.05, ‘.’—p < 0.1.

Table 2 shows the summary of the models for the modulation amplitudes of MAP and RRI. The “estimate” is an estimate of the coefficient beta of the predictor or the interaction of predictors in the linear model. The significant p-value of the predictor indicates that the predictor explains well some part of the variance in the model. Breathing frequency has a negative coefficient for both MAP and RRI, therefore with other factors controlled, the higher the frequency of breathing, the lower the amplitude of modulation. On the contrary, the addition of rotation (positive coefficient), increases the amplitude of modulation with respect to motionless conditions. For the RRI_A_, the Cone visual condition with no rotation also increases modulation.

Table 3 shows pairwise comparison contrasts for all conditions. The amplitudes of modulation of MAP are all significantly different between OVAR and Motionless conditions regardless of the visual stimulation. The amplitudes of modulation of RRI are all significantly different between OVAR conditions and Motionless Darkness and Cylinder conditions, but not between OVAR and Motionless Cone conditions. Moreover, the Motionless Cone condition is significantly different from the baseline Motionless Darkness condition.

**Table 3:** Pairwise contrasts for the LMM models for MAP_A_ and RRI_A_ (in the logarithmic scale) between all the conditions. P-value fdr-adjusted.

		MAP_A_				RRI_A_			
		Estimate	df	t-value	p-value	Estimate	df	t-value	p-value
Darkness motionless	Cylinder motionless	− 0.1069	186	− 0.535	0.6355	− 0.2183	186	− 1.405	0.2177
Darkness motionless	Cone motionless	− 0.1218	186	− 0.612	0.6249	− 0.4297	186	− 2.775	0.0130*
Darkness motionless	Darkness OVAR	− 3.1933	187	− 15.944	< 0.0001***	− 0.7497	187	− 4.815	< 0.0001***
Darkness motionless	Cylinder OVAR	− 3.3483	188	− 16.587	< 0.0001***	− 0.6687	188	− 4.260	0.0002**
Darkness motionless	Cone OVAR	− 2.9906	189	− 14.758	< 0.0001***	− 0.7201	188	− 4.570	0.0001**
Cylinder motionless	Cone motionless	− 0.0149	186	− 0.075	0.9406	− 0.2114	186	− 1.364	0.2177
Cylinder motionless	Darkness OVAR	− 3.0863	186	− 15.499	< 0.0001***	− 0.5314	186	− 3.433	0.0028**
Cylinder motionless	Cylinder OVAR	− 3.2414	186	− 16.228	< 0.0001***	− 0.4505	186	− 2.901	0.0104*
Cylinder motionless	Cone OVAR	− 2.8836	187	− 14.408	< 0.0001***	− 0.5018	187	− 3.225	0.0045**
Cone motionless	Darkness OVAR	− 3.0715	186	− 15.381	< 0.0001***	− 0.3200	186	− 2.062	0.0762
Cone motionless	Cylinder OVAR	− 3.2265	187	− 16.058	< 0.0001***	− 0.2391	187	− 1.530	0.1914
Cone motionless	Cone OVAR	− 2.8688	188	− 14.233	< 0.0001***	− 0.2904	188	− 1.853	0.1090
Darkness OVAR	Cylinder OVAR	− 0.1551	186	− 0.778	0.5472	0.0810	186	0.522	0.6947
Darkness OVAR	Cone OVAR	0.2027	186	1.015	0.4246	0.0296	186	0.191	0.8491
Cylinder OVAR	Cone OVAR	0.3578	186	1.797	0.1110	− 0.0514	186	− 0.332	0.7931

Signif. codes: ‘***’—p < 0.001, ‘**’—p < 0.01, ‘*’—p < 0.05, ‘.’—p < 0.1.

Motion sickness evaluation with the Graybiel’s diagnostic criteria has shown an average score of 4.5 ± 4.1 which corresponds to a moderate malaise^24^ in 36 subjects. No subjects included in the analysis were classified as having “frank sickness”, seven subjects were categorized as having a “severe sickness” (score > 7), 8 having a “moderate malaise A” (score 5–7), 6 having a “moderate malaise B” (score 3–4), 6 having a “slight malaise” (score 1–2), and 9 having no symptoms (score 0). Subjects presenting no symptoms or a “slight malaise” (n = 15; 9 male, 6 female participants) were put in the “not motion sick” group and those having a “moderate malaise or “severe sickness” (n = 21; 12 male, 9 female participants) were put in the “motion sick” group. The proportion of males and females in both groups was equal as confirmed by the Fisher’s chi^2^ test (p = 1). Mann–Whitney test showed no difference in the average RRI, average MAP, RRI_A_ or MAP_A_ between motion-sick and not motion-sick groups (p < 0.05 for all). Including the individual MS score in LMM has shown no significant effect of the MS score on the amplitudes of modulation of MAP (p = 0.95) or RRI (p = 0.29), so the factor was excluded from the final models. The duration of the experiment had no effect on the modulation amplitudes RRI_A_ and MAP_A_ and on the RRI, but affected the MAP (p < 0.001), pairwise post-hoc comparisons with fdr correction have shown a significant decrease in MAP during 5th and 6th trials compared to 1st (p < 0.001), 2nd (p < 0.05), and 3rd trials (p < 0.05) and of the 4th trial compared to the 1st (p < 0.05).

## Discussion

This study evaluated the effects of OVAR and visual stimulation on the modulation of the AP and HR. We found that OVAR induces significant modulations of MAP and HR, whereas for the visual stimulus, the cone induces significant modulations, but only of HR and differently if the subjects are rotating or not.

The higher peak of the MAP modulation cycle appears slightly before the nose-down position (Fig. 2) while the RR-interval is on the contrary lowest slightly before the nose-down position. This finding is coherent with the previous findings by Kaufmann et al.^2^ who demonstrated that AP and HR, as well as the muscle sympathetic nerve activity were entrained by OVAR, with the higher peak of AP and lower peak of RR-interval around the nose-down position. The modulation of the MAP by the OVAR could come from the difference in hydrostatic levels of the hand and heart in the nose-up and nose-down positions. However, these hydrostatic level differences were accounted for and canceled with the help of the dedicated system of Finometer Pro. In addition, the MAP modulation was in phase advance with the actual rotation of the body, and this phase shift was different between subjects, but quite persistent under different conditions for the same subject (Fig. 3). This observation suggests that the modulation is the result of neural regulation, as the hydrostatic phase would be the same for all subjects. Therefore, this study confirms previous findings that the vestibular stimulation by OVAR can modulate the AP and HR in healthy subjects^2^.

To analyze the effect of OVAR on the modulation of HR and AP, we needed to take into account another strong modulator affecting these parameters, which is the respiratory cycle. Breathing causes oscillations of the AP at the breathing frequency, as well as tachycardia during inspiration and bradycardia during expiration, the phenomenon called respiratory sinus arrhythmia. Breathing frequency in our data indeed is correlated to the modulation, and the closer the breathing frequency is to the stimulus frequency, the higher the modulation. Therefore, if respiration is not taken into account, the stimulus-related modulation would be blurred and its source could be misinterpreted, especially when the respiratory frequency is close to the stimulus frequency or its harmonics. Indeed, a previous study has demonstrated that OVAR is able to synchronize respiration in a subgroup of subjects^3^. To separate the respiratory effect on the modulation of physiological parameters, we have used the mixed model in the analysis of the data, where we correlated the individual amplitude of modulation of the MAP to the individual frequency of respiration. Having this parameter controlled, the visual and vestibular factors can therefore be assessed.

As for the MAP, modulation of the HR also presented individually varying phase shifts. The HR modulation amplitude was also strongly correlated with the breathing frequency, reflecting the respiratory sinus arrhythmia^28^. The HR was also modulated by the vestibular part of the stimulation.

### Visual-vestibular effect

Concerning the hypothesis that different visual reinterpretation of the same vestibular input would alter the autonomic response, the results are less clear. The MAP modulation was unaffected by the visual input. The HR modulation was affected by the Cone condition, but not by the Cylinder. This was expected, as long as it is the inclination part of the stimulus, which is absent in the Cylinder, that is supposed to provoke the adjustment of the AP. The HR was modulated differently from the control condition (Darkness/Immobile) when the Cone visual stimulus was projected while the subjects were immobile; however, when the OVAR stimulation was applied, no visual effect was discernible. It seems that the vestibular and visual effects on the HR do not add up independently, but that the vestibular effect overrides the visual one.

In sum, in normal subjects and in normal conditions, the HR can be modulated by visual cues, when only those are available to sense the motion. Moreover, only the motion that includes inclination with respect to gravitational vertical can modulate the HR. When cues from the vestibular system and the body are present, the visual input does not add any additional impact on the modulation. As all of those signals presumably are weighted and combined to form an integrated estimate of tilt, the weight of one signal might be greater than the others for certain types of regulations. The visual impact could also be diminished by the use of virtual reality goggles, which despite its immersiveness stays only an approximation of the natural visual environment. It has been shown that a sensory mismatch in such an internal representation of movement can lead to the rearrangement of the sensory modalities, by calibrating the modality which receives the lowest weight^29^. Also, the sense of presence when using VR varies between individuals^30,31^.

The fact that visual stimulus was able to modulate only the heart rate, but not the arterial pressure, may indicate that the autonomic response is driven by the parasympathetic system. Accordingly, recent data demonstrates parasympathetic activation by visual rotation in healthy male subjects^32^.

### MS and VIMS

OVAR is a relatively nauseogenic type of vestibular stimulation^33,34^. The procedure was designed to maximally avoid symptoms of MS but it was not possible to eliminate them completely. Therefore, the demonstration of a visual-autonomic effect may be limited by motion sickness which may interfere with the results. The use of virtual reality can also provoke Visually Induced Motion Sickness (VIMS)^35^. VIMS has been demonstrated to be associated with changes in autonomic functions such as modifications in skin conductance^36^ and an increase in heart rate^37^. VIMS symptoms vary with VR parameters such as image resolution, field of view, and scale factor^38^. The field of view above 60° seems to be more and more provocative^39^, so VIMS might be a potential contributor to motion sickness experienced by subjects in this study (field of view = 110°). These symptoms could modify the physiological parameters measured in the study.

However, the average values of AP or HR were stable across different conditions. This can be explained by the counterbalanced design of the study, where for different subjects each condition appears at a different time during the experiment, while the MS effects accumulate. Indeed, the order of trials had a significant effect on AP that decreased from the 1st trial to the last, which can be a sign of MS^40^. No difference between MS symptoms was found in male and female participants. Interestingly, no difference in AP or HR was found between participants reporting no symptoms or very slight symptoms of MS and those reporting moderate malaise or severe sickness according to Graybiel’s scale^24^.

Concerning the modulation of AP and HR, no difference between motion-sick and not motion sick groups has been found. Moreover, when including MS factor in the statistical model for modulation amplitudes, we found that it did not significantly affect the amplitudes of modulation of AP and HR. Indeed, the effect of MS would rather be reflected in average values, rather than affect modulation at the stimulus frequency.

### Multisensory integration

It needs to be noted that OVAR stimulates not only the vestibular sensors of gravity, the otolith system, but also the extra-vestibular graviceptors, located in the trunk by Mittelstaedt^41^. These sensors add to posture-related autonomic adaptations^3^ and perception of vertical^42^. Several studies have been dedicated to deducing the proportional effect of vestibular and trunk graviceptors in some functions: it has been found that eye movement is almost entirely controlled by the vestibular system^43^, while the autonomic functions and the perception of the vertical are affected by both gravity sensors^3,42^. However, vestibular graviceptors predominate for vertical perception^42^ while extra-vestibular graviceptors predominate for autonomic regulations^3^. The somatosensory system is also stimulated during OVAR and it is important for the perception of spatial orientation, as the loss of somatosensation raises the sensitivity threshold for body tilts^44^. We attempted to limit somatosensation by padding the subject, however it is impossible to completely eliminate the stimulation.

## Limitations

The major limitation of this study is the impossibility to avoid MS which could interfere with autonomic regulations. Evaluating MS after each stimulation might have been beneficial for assessing the impact of each type of stimulation on the AP and HR. However, it would not help to disentangle the possible effect of MS on the modulation of these parameters. Unfortunately, to date, there is no reliable method that can continuously and objectively assess MS, and its effects on AP or HR are not systematic^45^. The second limitation of this study is that real motion stimulates multiple sensory systems at once, therefore the observed effect should be attributed to the vestibular and trunk graviceptors, as well as the somatosensory system.

## Conclusion

Overall, we conclude that the graviceptors stimulation by OVAR modulates AP and HR. We also conclude that the visual stimulation that includes illusory body tilt can also modulate the HR, but not the AP, when only the visual stimulation is present. The hypothesis of the visual reinterpretation of the vestibular effect was not confirmed, as the visual effect disappears in presence of the real body rotation. Finally, we conclude that the vestibular and somatosensory effects on the HR modulation override the visual effects.

## Data Availability

The datasets used and/or analyzed during the current study available from the corresponding author on reasonable request.
